# Hepatic mRNA, microRNA, and miR-34a-Target responses in mice after 28 days exposure to doses of benzo(a)pyrene that elicit DNA damage and mutation

**DOI:** 10.1002/em.20668

**Published:** 2011-10

**Authors:** Amal I Malik, Andrew Williams, Christine L Lemieux, Paul A White, Carole L Yauk

**Affiliations:** Environmental Health Sciences and Research Bureau,Health Canada, 50 Colombine Driveway, Ottawa, Ontario, Canada K1A 0K9

**Keywords:** DNA adducts, LacZ mutation, toxicogenomics, Apoptosis, P53, MiR-34a

## Abstract

Benzo(a)pyrene (BaP) is a mutagenic carcinogen that is ubiquitous in our environment. To better understand the toxic effects of BaP and to explore the relationship between toxicity and toxicogenomics profiles, we assessed global mRNA and microRNA (miRNA) expression in Muta™Mouse. Adult male mice were exposed by oral gavage to 25, 50, and 75 mg/kg/day BaP for 28 days. Liver tissue was collected 3 days following the last treatment. Initially, we established that exposure to BaP led to the formation of hepatic DNA adducts and mutations in the *lac*Z transgene of the Muta™Mouse. We then analyzed hepatic gene expression profiles. Microarray analysis of liver samples revealed 134 differentially expressed transcripts (adjusted *P* < 0.05; fold changes > 1.5). The mRNAs most affected were involved in xenobiotic metabolism, immune response, and the downstream targets of p53. In this study, we found a significant 2.0 and 3.6-fold increase following exposure to 50 and 75 mg/kg/day BaP, respectively, relative to controls for miR-34a. This miRNA is involved in p53 response. No other significant changes in miRNAs were observed. The protein levels of five experimentally confirmed miR-34a targets were examined, and no major down-regulation was present. The results suggest that liver miRNAs are largely unresponsive to BaP doses that cause both DNA adducts and mutations. In summary, the validated miRNA and mRNA expression profiles following 28 day BaP exposure reflect a DNA damage response and effects on the cell cycle, consistent with the observed increases in DNA adducts and mutations. Environ. Mol. Mutagen., 2012. © 2011 Crown in the right of Canada

## INTRODUCTION

Benzo(a)pyrene (BaP) is a well-characterized environmental carcinogen that is a constituent of combustion emissions. BaP is metabolized by cytochrome P450s to generate two dozen metabolites, including BaP-7,8-diol-9,10-epoxide (BPDE) [Shimizu et al.,^2000^]. Early steps of BaP activation include binding to the aryl hydrocarbon receptor (AhR), leading to its translocation into the nucleus and subsequent heterodimerization with the AhR-nuclear translocator (Arnt). The active transcription factor composed of AhR and Arnt bind to xenobiotic-responsive elements in the promoters of various genes including a number of the cytochrome P450s, leading to the metabolic activation of BaP [Okey et al.,^1994^; Hankinson,^2005^; Nebert and Dalton,^2006^; Uno et al.,^2006^]. Since the discovery of the role of BPDE as the primary metabolite interacting with DNA by Sims et al. [1974], a large body of research has explored the mechanisms involved in BaP induced/mediated carcinogenicity. The bulk of this work demonstrates that BPDE forms DNA adducts that can ultimately result in G:C to A:T transition mutations, potentially leading to the initiation of tumor formation [Phillips,^1983^; Stansbury et al.,^1994^; Nebert and Dalton,^2006^].

The field of toxicogenomics provides an improved ability to elucidate the mechanisms of toxicological responses to BaP exposure, as well as reveal potentially novel modes of action and adverse health effects. Toxicogenomics studies generally examine global mRNA expression profiles following exposure to toxic chemicals, with the assumption that mRNA levels are correlated with protein abundance. These studies are sometimes imprecise as protein and mRNA levels are not always well correlated [Hudder and Novak,^2008^]. Post-transcriptional regulation by microRNAs (miRNAs) is one process that is implicated in this discrepancy. MiRNAs are small noncoding RNAs that are estimated to regulate up to 30% of all human genes [Sun and Tsao,^2008^; Wang et al.,^2008^]. MiRNAs regulate gene expression by binding to the 3′untranslated region of target mRNAs resulting in translation suppression or degradation of the target mRNA [Sun and Tsao,^2008^; Lynam-Lennon et al.,^2009^]. Since their discovery, miRNAs have been shown to play a role in virtually every cellular process, and have been associated with various diseases including cancer, Alzheimer's, viral infections, and endocrine disregulation (for review see [Hudder and Novak,^2008^] and [Singh et al.,^2008^]). In addition, various studies have argued for a major role for miRNAs in response to environmental stressors, chemicals, and toxins (reviewed in [Lema and Cunningham,^2010^]), as well as a role in the DNA damage response and in regulating enzymes involved in xenobiotic metabolism such as cytochrome P450s (reviewed in [Hudder and Novak,^2008^]). Thus, we hypothesize that exposure to a DNA-damaging chemical like BaP will alter both mRNAs and miRNAs associated with DNA damage, xenobiotic metabolism, and AhR signaling.

A challenge in profiling miRNAs is determining the appropriate exposure dose and time point for sampling. For example, Li et al. [2010] treated female Big Blue mice with a single acute dose (120 mg/kg) of the model genotoxic carcinogen *N*-ethyl-*N*-nitrosourea (ENU). The authors sampled 1, 3, 7, 15, 30, and 120 days after the exposure and found that hepatic miRNA expression changed over time, with a maximum response 7 and 15 days after the exposure. In contrast, Yauk et al. [2010] exposed male mice to a 150 mg/kg of another genotoxic carcinogen, BaP, for 3 days and found no evidence of changes in hepatic miRNAs 4 or 24 hr later, despite widespread changes in mRNA expression. Thus, while the data in the study suggest lack of induction of AhR-mediated miRNAs in mouse liver following BaP treatment, lack of miRNA response to DNA damage may be explained by insufficient postexposure time for the expression changes in miRNAs to be measurable.

This study examines the hypothesis that longer term exposure to highly mutagenic doses of BaP elicit liver toxicity and DNA damage in parallel with perturbations in mRNA and miRNA expression. We examined animals exposed to three doses of BaP (25, 50, and 75 mg/kg/day BaP) and expanded the exposure period to 28 days. The doses selected in this study were based on a literature review of transgenic rodent mutation assays; doses were selected that elicited a significant increase in transgene mutations in the liver [Lambert et al.,^2005^]. The Muta™ Mouse transgene mutation assay was used, and DNA adducts were quantified to confirm delivery of a mutagenic dose of BaP to the liver. We also assessed changes in selected proteins to determine if activated miRNAs play a role in mediating hepatic protein expression following murine BaP exposure. The results provide insight into the role of miRNAs in response to DNA adducts and mutation, and the mechanism of action of BaP.

## MATERIALS AND METHODS

### Animal Treatment

Male Muta™Mice (transgenic mouse strain 40.6; 25 weeks of age) were individually housed in a plastic film isolator (Harlan Isotec, UK) on a 12 hr light/12 hr dark cycle. Food (2014 Teklad Global standard rodent diet) and water were available ad libitum for the duration of the experiment. Animals were dosed daily via oral gavage for 28 days with BaP dissolved in olive oil (25, 50, and 75 mg/kg body weight/day). BaP was purchased from Sigma-Aldrich (Oakville, ON, Canada). Each dose group contained five animals; five animals were also dosed with olive oil as vehicle controls. Mice were sacrificed by cardiac puncture under isofluorane anaesthesia 3 days after the last dose. Liver was excised, flash-frozen in liquid nitrogen, and stored at −80°C until use. Mice were bred and maintained under conditions approved by the Health Canada Animal Care Committee.

### DNA Adduct Analysis

Genomic DNA was isolated from liver in order to examine the extent of DNA adduct formation. Liver tissue was thawed and homogenized on ice in 7 ml TMST buffer (50 mM Tris pH 7.6, 3 mM Magnesium Acetate, 0.2% (v/v), 250 mM Sucrose, 0.2% (v/v) Triton X-100). The liver homogenate was centrifuged for 6 min at 800 g (4°C), the supernatant was discarded, and the pellet was again washed twice with TMST buffer. The pellet was suspended in 5 ml lysis buffer (10 mM Tris pH 7.6, 10 mM EDTA, 150 mM NaCl, 1% (w/v) SDS and 1 mg/ml proteinase K (≥20 Units/mg). The liver homogenate was incubated overnight at 37°C. The following day, genomic DNA was isolated by phenol/choloroform extraction as described in [Douglas et al.,^1994^; Vijg and Douglas,^1996^]. Isolated DNA was dissolved in 100 μl TE buffer (10 mM Tris pH 7.6, 1 mM EDTA) and stored at 4°C until use.

DNA adducts were measured using the nuclease P1 digestion enrichment version of the ^32^P-postlabeling assay [Phillips and Arlt,^2007^]. All enzymes and chemicals used for this assay were purchased from sources previously described in [Phillips and Arlt,^2007^]. DNA (4 μg) was digested with micrococcal nuclease (120 mUnits) and calf spleen phosphodiesterase (40 mUnits), enriched with Nuclease P1, and labeled with [γ-^32^P]ATP as previously described [Phillips and Arlt,^2007^]. Enriched and labeled DNA samples were spotted onto polyethyleneimine-cellulose thin layer chromatography (TLC) plates and were separated chromatographically under the following conditions [Arlt et al.,^2008^]: D1, 1.0 M sodium phosphate, pH 6; D3, 4 M lithium-formate, 7 M urea, pH 3.5; D4, 0.8 M LiCl, 0.5 M Tris, 8.5 M urea, pH 8. TLC plates were scanned using a Packard Instant Imager (Dowers Grove, IL) and DNA adduct levels were calculated from the adduct cpm, the specific activity of ATP and the amount of DNA (pmol of DNA-P) used. A BaP diol-epoxide-DNA standard was used to identify B(a)P-DNA adducts [Phillips and Castegnaro,^1999^]. Results are expressed as DNA adducts/10^8^ nucleotides.

### LacZ Mutant Frequency

The frequency of *lacZ* transgene mutants in genomic DNA isolated from liver was measured using the P-Gal positive selection assay as previously described [Vijg and Douglas,^1996^; Lambert et al.,^2005^]. λgt10*lacZ* DNA was rescued from genomic DNA using the Transpack™ lambda packaging system (Stratagene, La Jolla, CA). Packaged phage particles were then mixed with the host bacterium (*Escherichia colilacZ^-^*, *galE^-^*, *recA^-^*, pAA119 with *galT* and *galK* [Gossen et al.,^1992^]), plated on minimal medium containing 0.3% (w/v) P-Gal and incubated overnight at 37°C. Total plaque-forming units (pfu) were measured on concurrent titers that did not contain P-Gal. Mutant frequency is expressed as the ratio of mutant pfu to total pfu. Mutant frequency data were analyzed using Poisson Regression in SAS v.9.1 (SAS Institute, Cary, NC). The data were fit to the model log(*E*(*Y*_*i*_)) = log *t*_*i*_ + íx*_i_*, where *E*(*Y*_*i*_) is the expected value for the *i*th observation, í is the vector of regressions coefficients, **x***_i_* is a vector of covariates for the *i*th observation, and *t*_*i*_ is the offset variable used to account for differences in observation count period (i.e., pfu). The offset (i.e., natural log of pfu) was given a constant coefficient of 1.0 for each observation, and log-linear relationships between mutant count and test article concentration were specified by a natural log link function. Type 1, or sequential analysis, was used to examine the statistical significance of the BaP treatment, and custom contrasts were used to evaluate the significance of responses at each dose.

### Total RNA Isolation

Total RNA was extracted as described previously [Yauk et al., 2010]. Briefly, total RNA was extracted from liver samples and purified using TRIzol reagent (Invitrogen, Carlsbad, CA) and RNeasy Mini Kits (Qiagen, Mississauga, ON, Canada), respectively. An RNase-Free DNase kit was used for DNase treatment according to the manufacturer's instructions (Qiagen, Mississauga, ON, Canada). All RNA samples showed A_260/280_ ratios between 2.0 and 2.1 and A_260/230_ ratios between 1.8 and 2.3. RNA integrity was also determined using an Agilent 2100 Bioanalyzer (Agilent Technologies, Mississauga, ON, Canada). Samples with RNA integrity numbers (RIN) between 7.5 and 9 were used for analysis.

### miRNA Isolation

Total RNA enriched in small RNA species was isolated from liver samples using mirVana miRNA Isolation kits (Ambion, Streetsville, ON, Canada) according to the manufacturer's recommendations. All RNA samples showed A_260/280_ ratios between 2.0 and 2.1 and A_260/230_ rations between 1.8 and 2.1. RNA integrity was also determined using an Agilent 2100 Bioanalyzer (Agilent Technologies, Mississauga, ON, Canada). Samples with RINs between 8.3 and 9.4 were used for analysis.

### mRNA Microarray Hybridization

Total RNA (200 ng) from 5 mice per treatment group (control, 25 mg/kg, 50 mg/kg, and 75 mg/kg) and universal reference total RNA (Stratagene, Mississauga, ON, Canada) were each used individually to synthesize double stranded cDNA. Agilent Linear Amplification kits and cyanine dye (Agilent Tech, Mississauga, ON, Canada) were used for the cDNA synthesis and cRNA labeling (treatment samples with Cyanine 5-CTP and reference RNA with Cyanine 3-CTP), respectively. The experiments were conducted according to the manufacturer's instructions. Cyanine-labeled cRNA targets were transcribed in vitro using T7 RNA polymerase and purified by RNeasy Mini Kit (Qiagen, Mississauga, ON, Canada). Hybridization was performed using the Agilent SureHyb hybridization chamber overnight at 60°C on Agilent mouse oligonucleotide microarrays (Agilent Technology, Mississauga, ON, Canada). Each slide contained four arrays with 45,220 features. Arrays were washed and scanned (using an Agilent G2505B scanner) according to the manufacturer's recommendations. Feature Extraction software version 10.7.3.1 (Agilent Technology) was used to extract the data.

### miRNA Microarray Hybridization

MirVana RNA samples (100 ng) from five control and five mice per treatment group (25, 50, and 75 mg/kg/day) were individually labeled using Agilent's miRNA complete labeling and Hybridization Kit (Agilent Tech, Mississauga, ON, Canada). Labeling, hybridization, and washing were conducted as described previously [Yauk et al., 2010]. Briefly, samples were dephosphorylated using calf intestinal phosphatase for 30 min at 37°C. Samples were then denatured using 100% DMSO before labeling with cyanine 3-pCp. Each labeled sample was hybridized on Agilent mouse miRNA array slides containing eight arrays with 15,744 features (Agilent Tech, Mississauga, ON, Canada). Hybridization was carried out overnight at 55°C. Arrays were washed according to manufacturer's protocol then scanned using an Agilent G2505B scanner. The data were extracted using Feature Extraction software version 10.7.3.1.

### Microarray Analysis

#### mRNA Expression Microarray Analysis

A reference design [Kerr and Churchill,^2001^, ^2007^] was used to analyze mRNA expression microarray data. The background fluorescence was measured using the negative control (-)3xSLv1 probes; probes with median signal intensities less than the trimmed mean (trim = 5%) plus three trimmed standard deviations of the (-)3xSLv1 probe were flagged as absent (within the background signal). Data were normalized using LOWESS [Yang et al.,^2002^] in R [reference]. Ratio intensity plots and heat maps for the raw and normalized data were constructed to identify outliers. One sample was removed from the analysis based on clustering. Differentially expressed transcripts (upregulated or downregulated relative to olive oil treated control animal liver samples) were determined using the MAANOVA library [Wu et al.,^2003^] in R. The statistical model included fixed effects of array and treatment condition and was applied to the log 2 of the absolute intensities. The Fs statistic [Cui et al.,^2005^] was used to test for treatment effects. The *P*-values for all statistical tests were estimated by the permutation method using residual shuffling, followed by adjustment for multiple comparisons using the false discovery rate (FDR) approach [Benjamini and Hochberg,^1995^]. The fold change calculations were based on the least-square means [Goodnight and Harvey,^1978^; Searle et al.,^1980^]. Significant genes were selected based on a FDR-adjusted *P*-value < 0.05 for any BaP exposed versus control contrast. Probes were considered present if at least four of the five samples within a condition had signal intensities greater than three trimmed SDs above the trimmed mean of the (-)3xSLv1 probes (background signal).

#### miRNA Microarray Analysis

Nonbackground subtracted raw data were cyclic lowess normalized [Bolstad et al.,^2003^]. Technical replicate probes were averaged using the median signal intensity. Boxplots and cluster analyses were used to identify potential outliers (i.e., poor quality chips). This quality control check resulted in the elimination of two arrays from the analysis. Identification of differentially expressed miRNAs was carried out at the probe level as well as the miRNA level (i.e., there were multiple replicate probes for the individual miRNAs). The MAANOVA model [Wu et al.,^2003^] included the slide as a block effect as each slide could accommodate eight samples. The Fs statistic [Cui et al.,^2005^] was used to test for differences between the controls and BaP treated samples. Permutation *P*-values obtained using residual shuffling were estimated and then FDR-adjusted [Benjamini and Hochberg,^1995^]. Least-square means were used to estimate the fold-change for each comparison. Significant miRNAs were selected using the 0.05 significance level for the FDR corrected p-values. Probes were considered present if at least four of five samples within a condition had signal intensities above the background. Cluster analysis of miRNA data was done using the Multivariate Analysis of microarray data using ADE4 [Culhane et al.,^2005^].

#### Pathway Specific RT-PCR Validation

P53 pathway focused mRNA expression profiling, using RT-PCR, was performed according to the manufacturer's recommendations (SABiosciences, Frederick, MD-cat#PAMM-027D). Total RNA (0.5 μg per sample) was reverse transcribed and real-time PCR was performed using a CFX96 real-time detection system (Bio-Rad, Mississauga, ON, Canada). *C*_t_ values for each well were normalized to the *Hprt1* reference mRNA. Transcripts were further normalized by subtracting the median delta *C*_t_ value for each sample. Differential expression was determined with a two sample bootstrap test [Higgins,^2004^] using the R software. The resulting *P*-values were then FDR-adjusted [Benjamini and Hochberg,^1995^], and the fold change was estimated using the ratio of the arithmetic mean of the treated samples to the mean of the control samples. Standard errors for the fold change values were estimated using the bootstrap test [Efron et al.,^1993^].

#### RT-PCR Validation of miRNA

The expression of miR-34a was validated using the Qiagen miScript PCR system. The procedure was performed according to the manufacturer's recommendations (Qiagen, Mississauga, ON, Canada). One μg total RNA (Ambion miRNA isolation) per sample was polyadenylated before reverse transcription, and real-time PCR was performed in duplicate for each sample using the CFX96 real-time detection system (Bio-Rad, Mississauga, ON, Canada). Expression levels of miR-34a were normalized to reference RNU1 expression. Statistical analysis of control and BaP treated samples was by Student's t-test.

#### Bioinformatics

All mRNA and miRNA microarray data (normalized and raw) have been deposited in the NCBI Gene Expression Omnibus database under accession number GSE24910. Following normalization, differentially expressed mRNAs were imported into Ingenuity Pathway Analysis (Ingenuity Systems, Redwood City, CA), the Database for Annotation, Visualization and Integrated Discovery, and KEGG pathway for identification of significantly enriched pathways.

#### Western Blot Analysis

Immunoblotting was performed as described previously [Malik and Storey,^2009^] with minor changes. Briefly, samples of frozen tissues were quickly weighed and homogenized 1:10 (w:v) with radioimmunoprecipitation assay buffer (Thermo Scientific, Rockford, IL) and a 1:1,000 (v/v) aliquot of Sigma Protease Inhibitor Cocktail (Sigma-Aldrich, Oakville, ON, Canada). Soluble protein concentration was quantified using the Pierce bovine serum albumin protein assay (Thermo scientific, Rockford, IL). Samples were then adjusted to a set protein concentration by dilution with RIPA buffer and mixed 1:2 (v/v) with SDS-PAGE loading buffer (Bio-Rad, Mississauga, ON, Canada). Equal amounts of protein were separated by SDS-PAGE on 10%–12% polyacrylamide gels and were transferred onto a polyvinylidene fluoride (Millipore, Billerica, MA) membrane by wet transfer. Membranes were probed with primary antibodies against Bcl2 (1:1,000; Cell Signaling Technology, Danvers, MA), Cyclin D1 (1:1,000; Cell Signaling Technology), β-actin (1:2,000; Cell Signaling Technology) E2f3 (1:400; Santa Cruz Biotechnology, Santa Cruz, CA), Cyclin E2 (1:400; Santa Cruz Biotechnology) and Cdk6 (1:400; Santa Cruz Biotechnology). Membranes were incubated with secondary antibody (1:20,000; Assay Designs, Ann Arbor, MI) for 30 min. Immunoreactive bands were visualized by enhanced chemiluminescence (Millipore Billerica, MA). Signals were quantified using image lab software version 3.0 build 11 (Bio-Rad, Mississauga, ON, Canada) and normalized relative to β-actin.

## RESULTS

Exposure to 25, 50, and 75 mg/kg/day BaP for 28 days caused no overt signs of toxicity in any of the animals on necropsy, and no animals exhibited significant weight loss compared with control (data not shown).

### DNA Adducts

**Fig 1:**
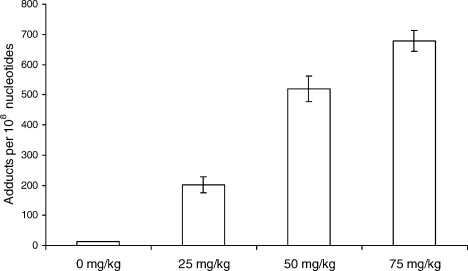
Relative Adduct Labeling (RAL) of DNA adducts measured in the liver of BaP-exposed Muta™Mouse. Error bars represent standard error of the mean of five mice.

All liver tissues obtained from BaP-exposed animals contained DNA adducts as measured using the ^32^P-postlabeling method. There was an overall effect of BaP dose on the level of DNA adducts (Fig 1; ANOVA *P* < 0.0001). Also, the level of adducts was significantly increased compared to control for all three dose groups (one-way Dunnett test, *P* < 0.05). The main adduct detected was identified as dG-N^2^-BPDE (BaP-7,8-diol-9,10-epoxide-N^2^-deoxyguanosine) using an external BaP diol-epoxide-DNA standard.

### LacZ Mutagenicity Assessment

**Fig 2:**
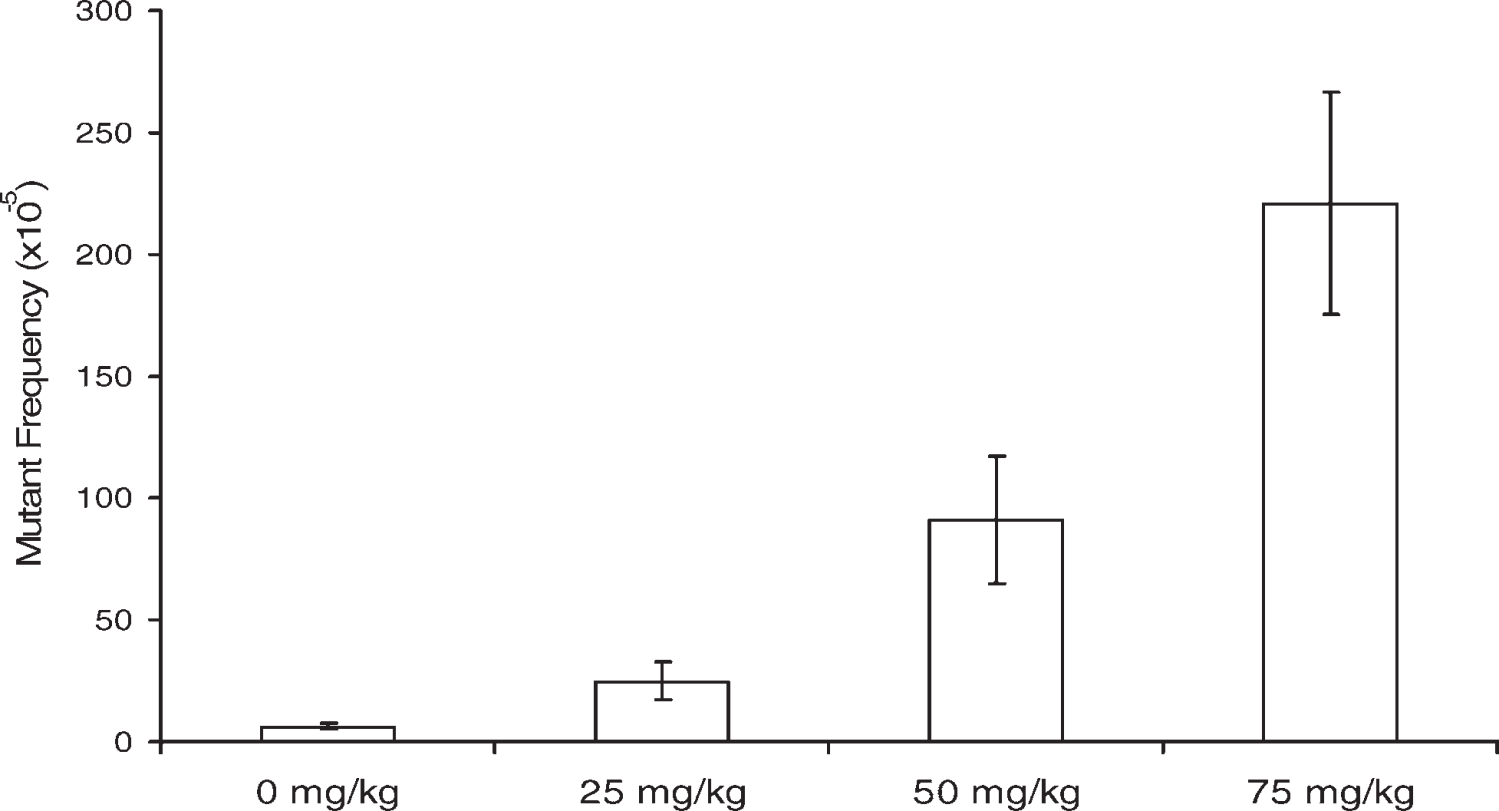
Mutant Frequency of the *lacZ* transgene in liver of BaP-treated Muta™Mouse. Error bars represent standard error of the mean of five mice.

A dose-dependent increase in mutant frequency was observed in the livers of BaP-treated animals (35.6-fold above control at the highest dose tested; Poisson regression chi-square analysis for BaP concentration effect = 237.95, *P* < 0.0001). Moreover, statistically significant increases in mutant frequency were observed at each BaP dose relative to control (Fig 2; *P* < 0.01). Pearson correlation analysis revealed a strong correlation between mutant frequency and DNA adducts (*r* > 0.964, *P* < 0.05). The DNA adduct and transgene mutation results confirmed effective delivery of BaP to the liver for all three doses examined.

### mRNA Expression

The two lowest doses elicited only minor changes in mRNA expression using our conservative statistical threshold. MAANOVA analysis revealed differential expression of two up-regulated and four down-regulated transcripts with fold-change > 1.5 (FDR-adjusted *P* < 0.05) in the 25 mg/kg/day BaP treatment group (Supporting Information Table I). Seven transcripts were up-regulated in response to 50 mg/kg/day BaP treatment (FDR-adjusted *P* < 0.05 and fold-change > 1.5). Exposure to the highest dose (75 mg/kg/day) had a major effect on mRNA expression with 105 up-regulated and 16 down-regulated genes with fold-change > 1.5 (FDR-adjusted *P* < 0.05; Supporting Information Table I). Many up-regulated mRNAs were involved in the metabolism of xenobiotics, including phase I and phase II metabolizing enzymes. The largest fold change (7.2-fold) was observed for cytochrome P450 3A44 (*Cyp3a44*). Other up-regulated cytochrome P450 family members included: *Cyp2b9, Cyp2c38*, and *Cyp2c40.* Enhanced expression of phase II metabolizing enzymes was also observed, including the up-regulation of glutathione S-transferase Mu 3, 4, and 7 (*Gstm3*, *Gstm4,* and *Gstm7*). A significant increase in the transcription of a number of p53 targets in response to 75 mg/kg/day BaP was also found (Supporting Information Table I). These changes included cyclin-dependent kinase inhibitor 1A (*Cdkn1a*), cyclin d1 (*Ccnd1*), cyclin g1 (*Ccng1*), zinc finger martin type 3 (*Zmat3*), and insulin-like growth fator-1 (*Igf1*). Analysis of significant mRNAs using hierarchical clustering revealed that samples of the same treatment group clustered together, except one sample in the 50 and 75 mg/kg BaP treatment groups (Supporting Information Fig 1). Thus, an overall treatment effect on gene expression was observed, even at the lowest doses. P53 pathway-focused expression profiling using RT-PCR arrays confirmed the response of many p53 targets and also identified additional responsive p53 targets in all three treatment groups that were not found by microarray analysis (Table I). In total, 11 transcripts (*P* < 0.05 and fold change > 1.5) of the 84 genes analyzed on the p53 pathway arrays were upregulated, and two were downregulated.

**Table I:** Validation of Microarray Results by Pathway Specific RT-PCR Array of Genes Related to p53-Mediated Signal Transduction

Accession number	Gene symbol	25 mg/kg				50 mg/kg						75 mg/kg							
		FDR *P*-value	Fold change	FDR *P*-value	Fold change	FDR *P*-value	Fold change
NM_007669	Cdkn1a	0.1875	1.9	0.0125	6.8	0.0040	16.9
NM_020275	Tnfrsf10b	0.1960	1.2	0.0025	3.7	0.0000	7.8
NM_007913	Egr1	0.4070	1.7	0.0475	4.1	0.0080	6.4
NM_007570	Btg2	0.4070	1.2	0.0195	2.2	0.0015	3.4
NM_009517	Zmat3	0.2910	1.2	0.0010	1.6	0.0035	2.4
NM_007630	Ccnb2	0.4895	1.1	0.1155	1.4	0.0010	2.4
NM_009684	Apaf1	0.0345	1.3	0.0055	1.8	0.0000	2.2
NM_145150	Prc1	0.2660	1.3	0.4235	1.2	0.0435	1.9
NM_009831	Ccng1	0.7125	−1.0	0.1125	1.2	0.0065	1.8
NM_009689	Birc5	0.7805	1.1	0.1770	1.4	0.0205	1.8
NM_007527	Bax	0.2230	1.1	0.1325	1.2	0.0000	1.7
NM_010866	Myod1	0.0375	−2.1	0.1000	−1.6	0.0970	−1.6
NM_011641	Trp63	0.0725	−2.1	0.0240	−2.5	0.2825	−1.5

Significantly expressed genes exhibiting fold change > 1.5 (FDR-adjusted *P*-value < 0.05) in at least 1 treatment group in response to 25, 50, and 75 mg/kg/day BaP. List is organized from largest to smallest fold change for the 75 mg/kg treatment group.

### miRNA and Protein Expression

**Fig 3:**
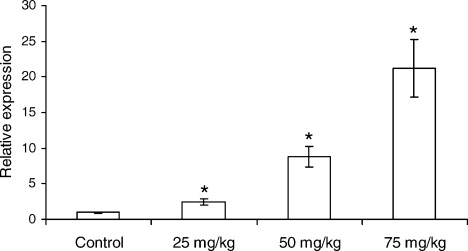
Relative miR-34a expression determined using the Qiagen miScript PCR system. * Significantly different from the corresponding control value (*n* = 4) using the Student's t-test, *P* < 0.05.

**Figure 4:**
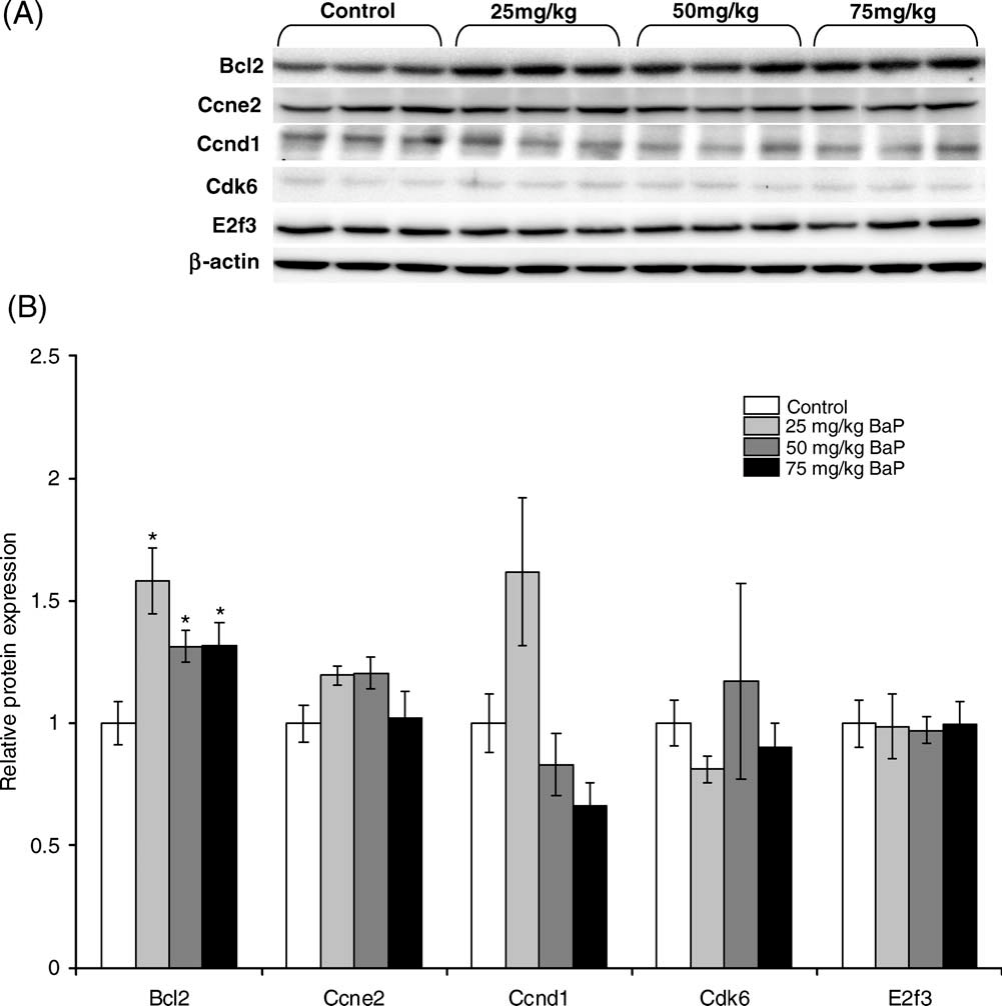
Western blot analysis of Bcl2, Ccne2, Ccnd1, Cdk6, and E2f3, protein expression in control, 25, 50, and 75 mg/kg/day BaP treated livers. (A) Representative Western immunoblot images showing levels of Bcl2, Ccne2, Ccnd1, Cdk6, E2f3, and β-actin in mouse liver using three independent samples. (B) Quantitative analysis of Western blots and summary histogram showing the expression of Bcl2, Ccne2, Ccnd1, Cdk6, and E2f3. Data are means ± S.E.M., *n* = 3 independent trials on tissue from x different animals. * Significantly different from the corresponding control value using the Student's t-test, *P*-value < 0.05.

Microarray analysis of miRNA expression revealed few changes in miRNA expression following BaP exposure, despite the presence of DNA adducts and large increases in mutation frequencies in the livers from these mice. Cluster analysis of miRNA data revealed no overall miRNA treatment response (Supporting Information Fig 2). The majority of the changes were very small (i.e., < 1.2-fold). Only one miRNA, miR-34a, exhibited a change in expression with FDR-adjusted *P*-value < 0.05 and fold change > 1.5 following treatment at the high doses (50 and 75 mg/kg BaP) (Table II). RT-PCR analyses also measured increased miR-34a levels in all BaP exposure groups relative to control (Fig 3). miR-34a has been identified as a downstream target of p53 and is implicated in regulating a number of p53 genes [Hermeking,^2007^]. To establish if miR-34a plays a role in the expression of p53 targets, we examined the mRNA and protein levels of 5 miR-34a targets that were experimentally confirmed in other studies: B-cell CLL/lymphoma 2 (Bcl2), cyclin-dependent kinase 6 (Cdk6), Ccnd1, CcnE2 and E2F3 [Chang et al.,^2007^; He et al.,^2007^; Hermeking,^2007^; Tazawa et al.,^2007^; Sun et al.,^2008^]. Our mRNA expression profiling showed a significant increase in the mRNA level of *Ccnd1* after BaP treatment but the expression of the other miRNA targets was not altered by BaP exposure (Supporting Information Table I). Western blot analysis showed a significant increase in Bcl2 protein levels after exposure to 25, 50, and 75 mg/kg/day BaP; no other significant changes in protein expression were observed (Fig 4).

**Table II:** DNA Microarray Data for miR-34 Expression in Response to 25, 50, and 75 mg/kg/day BaP

Dose (mg/kg)	FDR-adjusted *P*-value	Fold change
25	0.19	1.20
50	0.00	1.97
75	0.00	3.59

## DISCUSSION

BaP is an environmental carcinogen that induces DNA damage, gene mutations, and global changes in gene expression [Phillips,^1983^; Stansbury et al.,^1994^; Nebert and Dalton,^2006^]. Previous work has shown that despite the large disregulation of gene expression in liver following acute exposures to BaP, no significant changes in miRNA were found in the same liver samples [Yauk et al., 2010]. In this study, long-term (28 day) exposure to BaP resulted in the expected genetic toxicity response, with increases in DNA adducts (Fig 1) and *lacZ* transgene mutation (Fig 2). The primary adduct detected, dG-N^2^-BPDE, is well recognized as the main adduct induced by BaP metabolites [Cheng et al.,^1989^; Sayer et al.,^1991^]. Therefore, BaP reached the liver, was metabolically activated, induced DNA damage in the form of stable adducts, and induced transgene mutations. Thus, a genotoxic response was clearly manifested at the time of tissue collection. BaP treatment also activated the expected gene expression pathways including induction of DNA damage response, cell cycle arrest, apoptosis, immune response, inflammation, and apoptosis (see discussion below), and many of these genes exhibited a dose-response relationship. However, despite previous studies demonstrating the involvement of miRNAs in DNA damage response, hepatic miRNA changes, and changes in the production of selected proteins were minimal.

### Global mRNA Expression

Exposure to BaP for 28 days led to minor changes in mRNA expression at the two lowest doses (Supporting Information Table I). However, samples clustered by treatment group (Supporting Information Fig 1) demonstrating overall treatment effects even at the lowest dose. Thus, it is likely that additional genes were differentially expressed, but not identified in our analysis because of the conservative FDR-adjusted statistical threshold that we applied in an effort to minimize false positives. The high dose exposure caused a much larger effect on mRNA expression, with over 100 transcripts disregulated. A large proportion of these mRNAs are involved in phase 1 and 2 xenobiotic metabolism. However, given the presence of DNA adducts, the lack of upregulation of *cyp1a1* and *1b1* and the short half life (about 7 hr, [Uno et al.,^2004^; Arlt et al.,^2008^]) of BaP, we expect that the majority of the BaP had been metabolized in the liver at the time of necropsy, 3 days postexposure. Thus, a large proportion of the observed mRNA expression changes were likely responding to DNA damage, the presence of BaP metabolites, or other secondary effects. These expression changes provide evidence to support induction of DNA damage response, p53, tumor necrosis factor pathways, apoptosis, inflammatory, and immune response.

### P53 Pathway

Aside from the response in pathways and processes involved in phase 1 and phase 2 metabolism, microarray analysis revealed a significant effect of BaP on mRNAs in the p53 pathway. Transcriptional profiling by RT-PCR arrays confirmed changes in p53 responsive genes that are involved in cell cycle arrest, apoptosis, and p53 negative feedback (Table I). As the “guardian of the genome”, p53 play a central role in regulating a number of cellular process including cell cycle arrest, DNA repair, senescence, apoptosis, autophagy, and cell metabolism [Kruse and Gu,^2009^]. Lloyd and Hanawalt [Lloyd and Hanawalt,^2000^] also reported that p53 is required for repairing the DNA adducts formed by exposure to BPDE in human fibroblasts cells.

We did not find a significant increase in p53 mRNA expression following BaP treatment in this study. This is not surprising as p53 activation is generally achieved via a wide number of posttranslational modifications that include phosphorylation, methylation, acetylation, ubiquitination, and sumoylation [Lavin and Gueven,^2006^; Kruse and Gu,^2009^]. Activation and accumulation of p53 protein in vitro has been demonstrated after exposure to BaP and its metabolite BPDE [Pei et al.,^1999^; Lloyd and Hanawalt,^2000^; Park et al.,^2006^]. Park et al. [Park et al.,^2006^] showed the accumulation of p53 protein in as little as 3 hr following BaP treatment in human hepatoma HepG2 cells, while Pei et al. [1999] reported the accumulation of p53 protein after 8 and 24 hr of BaP treatment in A549 and NIH 3T3, respectively. Although no mRNA changes were observed for p53 itself following BaP exposure in this study, induction of downstream p53 targets suggests that p53 was activated.

Our data indicate that BaP exposure activates three distinct branches of the p53 pathway in the Muta™Mouse. First, BaP up-regulation of *Cdkn1a* mRNA is indicative of cell cycle arrest (Supporting Information Table I and Table I). *Cdkn1a* induction inhibits the formation of the CyclinE1/CDK2 complex, which prevents the downstream phosphorylation and inactivation of the retinoblastoma protein resulting in an inhibition of S phase and DNA synthesis (reviewed in [Caldon and Musgrove,^2010^]). Inducing growth arrest in this manner would allow the cell more time to repair DNA damage caused by BaP exposure [Kruse and Gu,^2009^]. This result is consistent with data published by Park et al. [2006] showing that p53 protein accumulation resulted in the up-regulation of *Cdkn1a* mRNA levels following BaP treatment. Indeed, upregulation of *Cdkn1a* is generally characteristic of a genotoxic stress response [Amundson et al.,^2005^; Ellinger-Ziegelbauer et al.,^2009^; Waters et al.,^2010^]. Second, the antiapoptotic branch of the p53 pathway is activated by BaP exposure, as evident by increases in the mRNAs for the protein regulator of cytokinesis 1 (*Prc1*), the inhibitor of apoptosis baculoviral IAP repeat-containing protein (*Birc-5*) [Altieri,^2003^; Shimo et al.,^2007^; Youle and Strasser,^2008^], and cyclin b 2 (*Ccnb2*) (Table I), which initiate the G2/M phase transition by binding to the mitosis promoting factor [Muller and Engeland,^2010^]. The third activated branch in the p53 network is apoptosis. Induction of apoptosis by BaP exposure is supported by increases in the expression of a number of pro-apoptotic mRNAs (Table I) including: (1) the caspase-9 activator apoptotic protease-activating factor-1 (*Apaf1*), (2) the pro-apoptotic Bcl-2 associated X (*Bax*) protein, (3) *Zmat3*; (4) *IGF*; (5) early growth response 1 (*Egr1*), and (6) B-cell translocation gene 2 (*Btg2*) [Krones-Herzig et al.,^2005^; Hata et al.,^2007^; Youle and Strasser,^2008^]. Thus, BaP exposure clearly resulted in the activation of p53 response pathway in the livers of exposed mice, indicative of a genotoxic stress and subsequent induction of cell cycle arrest and apoptotic/anti-apoptotic cellular responses.

The results above also support the findings of Amundson et al. [2005] that gene expression signatures can be used as an effective means to predict DNA damage response at the molecular level. Indeed, a number of the p53 responsive genes exhibited a dose-response relationship that parallels increasing DNA adduct and mutation frequencies (*Cdkn1a*, *Tnfrsf10b*, *Btg2*, *Apaf1*, and *Zmat*; Table I).

### P53 Independent Genes

A number of other pathways were induced in response to 28 day BaP exposure and, collectively, they provide molecular evidence for the induction of apoptosis, inflammation, immune response, and tissue injury.

Multiple mRNAs in the BaP expression profile from our microarray analysis suggest induction of inflammatory responses. For example, the highest BaP dose led to transcriptional up-regulation of three members of the tumour necrosis factor receptor super-family (TNFRSF): (1) nerve growth factor receptor (*Ngfr*), (2) *Tnfrsf1a*, (3) *Tnfrsf10b* (Supporting Information Table I; Table I). The main function of most TNFRSF members is to respond to ligand binding by activating different signal transduction pathways, most of which lead to apoptosis (reviewed in [Ashkenazi,^2002^]). Potential initiation of an acute inflammatory response is suggested by the significant increase of interleukin 1 beta (*Il-1b*) [Feghali and Wright,^1997^] in the 75 mg/kg/day BaP treatment group (Supporting Information Table I). Upregulation of leukotriene C4 synthase (*Ltc4s*) is also of interest (Supporting Information Table I). The product of *Ltc4s* catalyzes the conjugation of leukotriene A4 with reduced glutathione to form leukotriene C4, a potent proinflammatory lipid mediator synthesized from arachidonic acid [Uhlig and Wendel,^1992^; Lam et al.,^1994^]. The mRNA and protein expression of *Ltc4s* are both up-regulated in response to ischemia-reperfusion injury in hepatocytes and sinusoidal endothelial cells in rat [Yang et al.,^2007^]. In this study, *Ltc4s* exhibited a dose dependent increase in expression, which might be indicative of the initiation of liver injury at the medium and high doses. Thus, several genes in the hepatic expression profiles following BaP exposure are indicative of an inflammatory response and liver injury.

The gene expression changes also support the induction of an immune response following BaP treatment. This observation extends to both the high and low doses of BaP. For example, the immune system responsive genes macrophage receptor with collagenous structure (*Marco*) and immunoglobulin heavy chain 6 (*Igh-6*) [Kraal et al.,^2000^; Mastroeni et al.,^2000^] were significantly upregulated in response to 50 mg/kg BaP (Supporting Information Table I). Exposure to 25 mg/kg BaP led to the upregulation of ADAM-like, decysin 1 (*Adamdec1)*, a gene known to be expressed by dendritic cells and macrophages that is implicated in dendritic cell function [Bates et al.,^2002^] (Supporting Information Table I). The significant over-expression of extra cellular link domain-containing 1 (*Xlkd1*), known as lymphatic vessel endothelial hyaluronan receptor, was also observed. *Xlkd1* is abundant in lymphatic endothelium and is present at lower levels in activated tissue macrophages in the sinusoidal endothelium of liver. Xlkd1 plays a role in lymphatic hyaluronan transport and may be involved in tumor metastasis (for review; see [Jackson,^2003^]). The upregulation of the genes described above was unexpected due to the known immunosuppression effects of BaP ([De Jong et al.,^1999^; Schellenberger et al.,^2009^]. Perturbations of hepatic genes involved in immune response may play a role in this process. However, the direct relationship between the upregulation of these mRNAs and immunosuppression is unclear at this time.

The data support the notion that gene expression profiles may be useful indicators of molecular events arising at early time points that provide information on initiating events leading to chronic effects. However, additional experiments in animals following chronic and sub-chronic exposures to BaP in parallel with measured inflammatory and immune responses at later time points are necessary to support this hypothesis.

### MiR-34a and Protein Expression

Previously we showed that short term exposure (3 days) to high doses of BaP had very little effect on hepatic miRNA expression [Yauk et al., 2010]. In this study, we demonstrated that hepatic expression of only one miRNA, miR-34a, was increased significantly by sub-chronic BaP treatment at relatively high doses (Table II; Fig 3). Thus, the presence of bulky BaP DNA adducts and induction of mutation in the livers of these mice had little impact on miRNA expression, even after 28 days BaP exposure.

Recently, several laboratories have reported the discovery of the miR-34 family of miRNAs, which are known to be direct downstream targets of p53 [Hermeking,^2007^]. The family encompasses three known members. The first is miR-34a, which is encoded by its own transcript, is ubiquitously expressed, and is commonly deleted in human cancers [Chang et al.,^2007^; Hermeking,^2007^]. The other two members are miR-34b and miR-34c, which share a common primary transcript [Hermeking,^2007^]. MiR-34a, the only differentially regulated miRNA in this study, is contained within the second exon of an expressed sequence tag that has a p53 binding site located ˜30 kb upstream of the mature miR-34a [Chang et al.,^2007^; Raver-Shapira et al.,^2007^]. MiR-34a is highly upregulated following p53 activation [Chang et al.,^2007^; Hermeking,^2007^] and to date has been demonstrated to be involved in: (a) cell cycle arrest in the G1 phase; (b) induction of cell senescence; and (c) apoptosis [Chang et al.,^2007^; He et al.,^2007^; Hermeking,^2007^]. Recent studies have also shown that miR-34a may be required for p53 mediated tumour suppression [Hermeking,^2007^]. Thus, it has been proposed that miR-34a plays an important role in the modulation of the gene expression program initiated by p53.

MiR-34a induction may play a role in the post-transcriptional regulation of p53 mRNA targets without requiring the translation of additional effector proteins. By doing so, irreversible responses of p53 activation may be mediated [Hermeking,^2007^]. Recently, Guo et al. [2010] demonstrated that miRNAs exert their impact on gene expression mainly by destabilizing their target mRNAs or blocking translation. In an effort to pinpoint the role of miR-34a in the response to sub-chronic BaP treatment, we focused on the mRNA expression of 5 known miR-34a downstream targets: *Bcl2, Ccnd1, Ccne2*, *Cdk6,* and *E2f3*. Our microarray results indicate no significant decrease in their mRNA levels in any BaP treatment group (Supporting Information Table I; Table I). In addition to being targets of miR-34a, three of these genes (*Ccnd1*, *Ccne2*, *Cdk6*) are also negatively regulated by Cdkn1a whose mRNA expression was increased after BaP treatment in our study. In contrast, the level of *Ccnd1* mRNA actually increased 2.2 fold in response to 75 mg/kg/day BaP treatment (by microarray analysis, Supporting Information Table I). We also used Western blotting to assess the protein levels of the same targets in an effort to determine if miR-34a exerted its action through decreasing the translation efficiency of these transcripts. No significant down regulation of protein levels were observed for any of the tested targets (Fig 4). Evidence of increased mRNA for *Ccnd1* in the absence of an increase in the protein level may be the result of translational repression mediation by miR-34a. Other studies have suggested that the rapid degradation of *Ccnd1* following environmental stress or DNA damage could be a means of rapid cell cycle arrest to insure DNA integrity [Alao,^2007^]. However, because of the cyclical nature of cyclins, additional time points will be necessary to study this response in detail. It is clear that complex multifactorial mechanisms are at work in controlling the cyclin response to DNA damage.

Contrary to our expectations, Bcl2 protein level was significantly higher in the three treatment groups compared with controls. Although translation of some proteins can be upregulated by miRNA under special conditions [Vasudevan et al.,^2007^; Mott,^2009^], we speculate that the upregulation of Bcl2 could support the survival of cells in the face of a DNA damage response since the main function of Bcl2 overexpression is to inhibit cell death [Youle and Strasser,^2008^]. We also note that it is possible that in the absence of miR-34a, Bcl2 protein levels may be even more elevated (i.e., miR-34a is regulating Bcl2 but not to an extent that would lead to its levels being below control samples). The mechanism by which Bcl2 protein is upregulated is unclear; sampling additional time points throughout pre- and postexposure to examine miR-34a and Bcl2 levels, and experiments examining the effects of miR-34a overexpression or suppression in the presence and absence of BaP would shed light on the complex interactions between miR-34a, its target genes, and DNA damage response.

MiRNA studies in *Caenorhabditiselegans* have demonstrated that one or several miRNAs can regulate biological processes by targeting specific genes in the same pathway [He et al.,^2007^]. In this study, we did not detect any significant decreases in the mRNA or protein of any of the targets tested. Analysis in silico for targets of miR-34a by TargetScan [Friedman et al.,^2009^] revealed 394 potential targets. Only one of these targets (protein tyrosine phosphatase, receptor type, M; *Ptprm*) was significantly downregulated (1.4-fold; microarray analysis) following treatment with 75 mg/kg BaP. This gene is thought to be involved in signal transduction and may play a role in cell growth. However, it is clear that this work was unable to clearly demonstrate downregulation of any miR-34a targets. The reasons for the lack of effects on downstream targets may include: (1) miR-34a is not actively binding to its targets in the experimental conditions; (2) miR-34a is targeting another pathway that has not been discovered; (3) there might be a delayed response to miR-34a that was not encompassed in the time point studied; or (4) miR-34a is actively regulating its target genes, but levels of mRNAs and proteins are not suppressed below the control levels.

## CONCLUSION

We demonstrate that 28-day oral exposure to BaP resulted in a dose dependent increase in BPDE DNA adducts, and stable mutations in the *lacZ* transgene of Muta™Mouse. Analysis of global transcription showed up-regulation of xenobiotic metabolism, immune response, DNA damage response, cell cycle arrest, antiapoptotic, and apoptotic genes. Analysis of miRNAs revealed significant upregulation of miR-34a. This result is consistent with a DNA damage response induced through the p53 signaling pathway and supports the notion that induction of specific DNA damage response genes can be used to identify a potentially mutagenic mode of action using DNA microarrays. However, we found measurable levels of BaP adducts at doses below those that induced significant changes in p53 DNA damage response genes (e.g., *Cdkn1a*). This finding is in keeping with those of Akerman et al. [2004]; they exposed TK6 cells in culture to BPDE and found that DNA adduct formation was a more sensitive indicator of exposure than gene expression assessed using DNA microarrays. These authors suggest that expression profiles for DNA damage response are more robust at toxic doses where mutations were evident.

This study clearly shows a lack of global hepatic miRNA response in mice exposed to BaP for 28 days. In the presence of high levels of DNA adducts and doses that led to the induction of DNA sequence mutations, only miR-34a was significantly altered. Thus, these results, in combination with our previous findings, indicate that the effects of hepatic miRNAs on response to BaP are minimal.

MiR-34a expression was significantly elevated (as measured by RT-PCR) even at the lowest dose of BaP, suggesting it may be a sensitive indicator of p53 response to DNA damage. In addition, miR-34a exhibited a dose-response that was similar to DNA adduct and mutation levels. However, analysis failed to identify directly responsive biological targets of miR-34a at the mRNA or protein level. Future work should aim to clarify the role of miR-34a in response to mutagenic agents. This work should include analysis of miR-34a response following exposure to mutagens with different modes of action, as well as protein and mRNA changes in cell cultures in the presence (over-expressed) or absence (silenced) of miR-34a. These experiments should focus on the role of miR-34a in DNA damage response, DNA repair, cell cycle, and apoptosis.
