# Supplementary material for: Hepatic mRNA, microRNA, and miR-34a-Target responses in mice after 28 days exposure to doses of benzo(a)pyrene that elicit DNA damage and mutation
Source: Environ Mol Mutagen. 2011 Oct;53(1):10–21. doi: 10.1002/em.20668 (PMC3525943; doi:10.1002/em.20668)

**Supplementary Fig. 1.**

The heat map shows the correlation among samples for genes that were statistically differentially expressed with a fold change above 1.5 fold. Red bars represent high expression levels relative to the reference sample, green bars represent low expression levels and black bars are similar to the normalized median gene expression values. The samples are clustered by treatment.


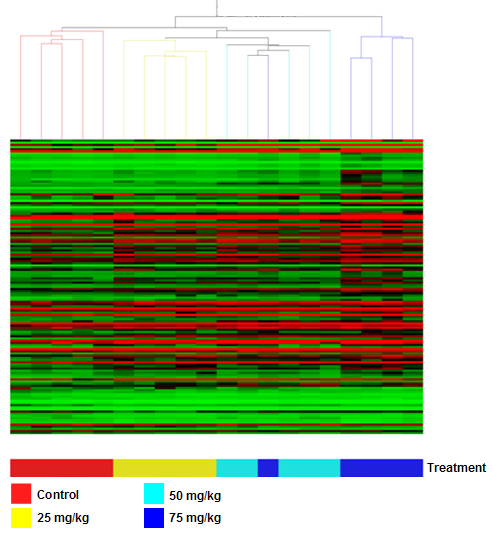

Supplement: Supplementary file 1 [file em0053-0010-SD1.doc]
