# Supplementary material for: Hepatic mRNA, microRNA, and miR-34a-Target responses in mice after 28 days exposure to doses of benzo(a)pyrene that elicit DNA damage and mutation
Source: Environ Mol Mutagen. 2011 Oct;53(1):10–21. doi: 10.1002/em.20668 (PMC3525943; doi:10.1002/em.20668)
Supplement: Supplementary file 2 [file em0053-0010-SD2.doc]

**Supplementary Table 1.**

Summary of all significantly expressed genes exhibiting fold change > 1.5 (FDR-adjusted p-value < 0.05) in at least 1 treatment group in response to 25, 50, and 75 mg/kg/day BaP. List is organized from largest to smallest fold change for the 75 mg/kg treatment group. Members of the p53 signaling pathway are presented in blue.

|  | |  | | |  | | |  | | | **25 mg/kg** | | | | | **50 mg/kg** | | | | | **75 mg/kg** | | | | |
| --- | --- | --- | --- | --- | --- | --- | --- | --- | --- | --- | --- | --- | --- | --- | --- | --- | --- | --- | --- | --- | --- | --- | --- | --- | --- |
|  | **GenBank** | | | **GENE_SYMBOL** | | | **DESCRIPTION** | | | **Adjusted p-value** | | | **Fold change** | | **Adjusted p-value** | | | **Fold change** | | **Adjusted p-value** | | | **Fold change** | |  |
| NM_177380 | | | Cyp3a44 | | | Mus musculus cytochrome P450, family 3, subfamily a, polypeptide 44 (Cyp3a44) | | |  | | |  | |  | | |  | | 0.0000 | | | 7.22 | |  | |
| NM_010766 | | | Marco | | | Mus musculus macrophage receptor with collagenous structure (Marco) | | |  | | |  | | 0.0000 | | | 3.69 | | 0.0000 | | | 5.21 | |  | |
| NM_008521 | | | Ltc4s | | | Mus musculus leukotriene C4 synthase (Ltc4s) | | |  | | |  | | 0.0361 | | | 1.96 | | 0.0000 | | | 4.43 | |  | |
| NM_053247 | | | Xlkd1 | | | Mus musculus extra cellular link domain-containing 1 (Xlkd1) | | |  | | |  | |  | | |  | | 0.0000 | | | 3.55 | |  | |
| BC058107 | | | 2410089E03Rik | | | Mus musculus RIKEN cDNA 2410089E03 gene | | |  | | |  | |  | | |  | | 0.0000 | | | 3.54 | |  | |
| NM_011575 | | | Tff3 | | | Mus musculus trefoil factor 3, intestinal (Tff3) | | |  | | |  | |  | | |  | | 0.0000 | | | 3.17 | |  | |
| AK033690 | | | 9130221J18Rik | | | Mus musculus adult male cecum cDNA, RIKEN full-length enriched library | | |  | | |  | |  | | |  | | 0.0079 | | | 3.06 | |  | |
| NM_010924 | | | Nnmt | | | Mus musculus nicotinamide N-methyltransferase (Nnmt) | | |  | | |  | |  | | |  | | 0.0275 | | | -2.98 | |  | |
| NM_009114 | | | S100a9 | | | Mus musculus S100 calcium binding protein A9 (calgranulin B) (S100a9) | | |  | | |  | |  | | |  | | 0.0079 | | | 2.92 | |  | |
| NM_027040 | | | 1700007K13Rik | | | Mus musculus RIKEN cDNA 1700007K13 gene | | |  | | |  | |  | | |  | | 0.0000 | | | 2.85 | |  | |
| NM_007669 | | | Cdkn1a | | | Mus musculus cyclin-dependent kinase inhibitor 1A (P21) (Cdkn1a) | | |  | | |  | |  | | |  | | 0.0000 | | | 2.76 | |  | |
| NM_030143 | | | Ddit4l | | | Mus musculus DNA-damage-inducible transcript 4-like (Ddit4l) | | |  | | |  | |  | | |  | | 0.0070 | | | 2.57 | |  | |
| NM_013809 | | | Cyp2g1 | | | Mus musculus cytochrome P450, family 2, subfamily g, polypeptide 1 (Cyp2g1) | | |  | | |  | | 0.0000 | | | 2.30 | | 0.0000 | | | 2.76 | |  | |
| NM_011254 | | | Rbp1 | | | Mus musculus retinol binding protein 1, cellular (Rbp1) | | |  | | |  | |  | | |  | | 0.0088 | | | 2.62 | |  | |
| NM_001013753 | | | Pcdh17 | | | Mus musculus protocadherin 17 (Pcdh17) | | |  | | |  | |  | | |  | | 0.0000 | | | 2.59 | |  | |
| NM_153127 | | | Mmrn2 | | | Mus musculus multimerin 2 (Mmrn2) | | |  | | |  | | 0.0000 | | | 1.71 | | 0.0000 | | | 2.57 | |  | |
| XM_981410 | | | 9030619P08Rik | | | PREDICTED: Mus musculus RIKEN cDNA | | |  | | |  | |  | | |  | | 0.0173 | | | 2.44 | |  | |
| NM_054088 | | | Pnpla3 | | | Mus musculus patatin-like phospholipase domain containing 3 (Pnpla3) | | |  | | |  | |  | | |  | | 0.0337 | | | 2.43 | |  | |
| NM_177615 | | | C78409 | | | Mus musculus expressed sequence C78409 (C78409) | | |  | | |  | |  | | |  | | 0.0000 | | | 2.42 | |  | |
| NM_011388 | | | Slc10a2 | | | Mus musculus solute carrier family 10, member 2 (Slc10a2) | | |  | | |  | |  | | |  | | 0.0079 | | | 2.35 | |  | |
| NM_009160 | | | Sftpd | | | Mus musculus surfactant associated protein D (Sftpd) | | |  | | |  | |  | | |  | | 0.0079 | | | 2.29 | |  | |
| BC063325 | | | Serpina3a | | | Mus musculus serine (or cysteine) peptidase inhibitor, clade A, member 3A | | |  | | |  | |  | | |  | | 0.0433 | | | 2.27 | |  | |
| NM_008902 | | | Pp11r | | | Mus musculus placental protein 11 related (Pp11r) | | |  | | |  | |  | | |  | | 0.0000 | | | 2.21 | |  | |
| NM_007631 | | | Ccnd1 | | | Mus musculus cyclin D1 (Ccnd1), mRNA [NM_007631] | | |  | | |  | |  | | |  | | 0.0000 | | | 2.13 | |  | |
| NM_197985 | | | Adipor2 | | | Mus musculus adiponectin receptor 2 (Adipor2) | | |  | | |  | |  | | |  | | 0.0449 | | | 2.18 | |  | |
| NM_027102 | | | Esam1 | | | Mus musculus endothelial cell-specific adhesion molecule (Esam1) | | |  | | |  | |  | | |  | | 0.0079 | | | 2.13 | |  | |
| NM_010000 | | | Cyp2b9 | | | Mus musculus cytochrome P450, family 2, subfamily b, polypeptide 9 (Cyp2b9) | | |  | | |  | |  | | |  | | 0.0133 | | | 2.13 | |  | |
| NM_025915 | | | Tmem88 | | | Mus musculus transmembrane protein 88 (Tmem88) | | |  | | |  | |  | | |  | | 0.0079 | | | 2.12 | |  | |
| NM_009831 | | | Ccng1 | | | Mus musculus cyclin G1 (Ccng1) | | |  | | |  | |  | | |  | | 0.0133 | | | 2.12 | |  | |
| NM_021301 | | | Slc15a2 | | | Mus musculus solute carrier family 15 (H+/peptide transporter), member 2 (Slc15a2) | | |  | | |  | |  | | |  | | 0.0133 | | | 2.10 | |  | |
| BC066148 | | | 4632434I11Rik | | | Mus musculus RIKEN cDNA 4632434I11 gene | | |  | | |  | |  | | |  | | 0.0000 | | | 2.04 | |  | |
| AK044205 | | | A930001C03Rik | | | Mus musculus RIKEN cDNA A930001C03 gene | | |  | | |  | |  | | |  | | 0.0000 | | | 2.01 | |  | |
| NM_144869 | | | BC021614 | | | Mus musculus cDNA sequence BC021614 (BC021614) | | |  | | |  | |  | | |  | | 0.0248 | | | 1.97 | |  | |
| NM_008685 | | | Nfe2 | | | Mus musculus nuclear factor, erythroid derived 2 (Nfe2) | | |  | | |  | |  | | |  | | 0.0337 | | | 1.95 | |  | |
| AK041703 | | | 2410089E03Rik | | | 2410089E03Rik RIKEN cDNA 2410089E03 gene | | |  | | |  | |  | | |  | | 0.0000 | | | 1.94 | |  | |
| NM_008963 | | | Ptgds | | | Mus musculus prostaglandin D2 synthase (brain) (Ptgds) | | |  | | |  | |  | | |  | | 0.0079 | | | 1.94 | |  | |
| NM_153175 | | | Gimap6 | | | Mus musculus GTPase, IMAP family member 6 (Gimap6) | | |  | | |  | |  | | |  | | 0.0388 | | | 1.94 | |  | |
| NM_011388 | | | Slc10a2 | | | Mus musculus solute carrier family 10, member 2 (Slc10a2) | | |  | | |  | |  | | |  | | 0.0133 | | | 1.89 | |  | |
| W15861 | | | Isg20l1 | | | mb53g10.r1 Soares mouse p3NMF19.5 | | |  | | |  | |  | | |  | | 0.0000 | | | 1.89 | |  | |
| D86232 | | | Ly6c | | | Mus musculus mRNA for Ly-6C variant, complete cds. [D86232] | | |  | | |  | |  | | |  | | 0.0000 | | | 1.88 | |  | |
| NM_016886 | | | Gria3 | | | Mus musculus glutamate receptor, ionotropic, AMPA3 (alpha 3) (Gria3) | | |  | | |  | |  | | |  | | 0.0000 | | | 1.88 | |  | |
| NM_013750 | | | Phlda3 | | | Mus musculus pleckstrin homology-like domain, family A, member 3 (Phlda3) | | |  | | |  | |  | | |  | | 0.0000 | | | 1.87 | |  | |
| NM_029310 | | | 1700008G05Rik | | | Mus musculus RIKEN cDNA 1700008G05 gene (1700008G05Rik) | | |  | | |  | |  | | |  | | 0.0275 | | | 1.85 | |  | |
| NM_010002 | | | Cyp2c38 | | | Mus musculus cytochrome P450, family 2, subfamily c, polypeptide 38 (Cyp2c38) | | |  | | |  | |  | | |  | | 0.0079 | | | 1.84 | |  | |
| XM_283438 | | | Gpr116 | | | PREDICTED: Mus musculus G protein-coupled receptor 116, transcript variant 1 (Gpr116) | | |  | | |  | |  | | |  | | 0.0000 | | | 1.84 | |  | |
| NM_015784 | | | Postn | | | Mus musculus periostin, osteoblast specific factor (Postn) | | |  | | |  | |  | | |  | | 0.0133 | | | 1.83 | |  | |
| NM_033217 | | | Ngfr | | | Mus musculus nerve growth factor receptor (TNFR superfamily, member 16) (Ngfr) | | |  | | |  | |  | | |  | | 0.02 | | | 1.83 | |  | |
| AK029268 | | | Eda2r | | | Mus musculus Eda2r ectodysplasin A2 receptor | | |  | | |  | |  | | |  | | 0.0079 | | | 1.82 | |  | |
| NM_026018 | | | Pdzk1ip1 | | | Mus musculus PDZK1 interacting protein 1 (Pdzk1ip1) | | |  | | |  | |  | | |  | | 0.0000 | | | 1.81 | |  | |
| NM_010359 | | | Gstm3 | | | Mus musculus glutathione S-transferase, mu 3 (Gstm3) | | |  | | |  | |  | | |  | | 0.0000 | | | 1.81 | |  | |
| NM_172621 | | | Clic5 | | | Mus musculus chloride intracellular channel 5 (Clic5) | | |  | | |  | |  | | |  | | 0.0000 | | | -1.81 | |  | |
| NM_011125 | | | Pltp | | | Mus musculus phospholipid transfer protein (Pltp) | | |  | | |  | |  | | |  | | 0.0000 | | | 1.81 | |  | |
| NM_010494 | | | Icam2 | | | Mus musculus intercellular adhesion molecule 2 (Icam2) | | |  | | |  | |  | | |  | | 0.0000 | | | 1.79 | |  | |
| NM_008882 | | | Plxna2 | | | Mus musculus mRNA for mKIAA0463 protein [AK122289] | | |  | | |  | |  | | |  | | 0.0224 | | | 1.79 | |  | |
| NM_207105 | | | H2-Ab1 | | | Mus musculus histocompatibility 2, class II antigen A, beta 1 (H2-Ab1) | | |  | | |  | | 0.0000 | | | 1.64 | | 0.0004 | | | 1.78 | |  | |
| NM_172759 | | | Ces5 | | | Mus musculus carboxylesterase 5 (Ces5) | | |  | | |  | |  | | |  | | 0.0248 | | | 1.78 | |  | |
| NM_026467 | | | Rps27l | | | Mus musculus ribosomal protein S27-like (Rps27l) | | |  | | |  | |  | | |  | | 0.0079 | | | 1.77 | |  | |
| NM_021475 | | | Adamdec1 | | | Mus musculus ADAM-like, decysin 1 (Adamdec1) | | | 0.0000 | | | 2.94 | |  | | |  | |  | | |  | |  | |
| AK088666 | | | Igh-6 | | | Mus musculus immunoglobulin heavy chain 6 (heavy chain of IgM) | | |  | | |  | | 0.0000 | | | 3.19 | |  | | |  | |  | |
| BC058107 | | | 2410089E03Rik | | | Mus musculus RIKEN cDNA 2410089E03 gene | | |  | | |  | |  | | |  | | 0.0000 | | | 1.75 | |  | |
| NM_175494 | | | Zfp367 | | | Mus musculus zinc finger protein 367 (Zfp367) | | |  | | |  | |  | | |  | | 0.0337 | | | -1.75 | |  | |
| NM_022315 | | | Smoc2 | | | Mus musculus SPARC related modular calcium binding 2 (Smoc2) | | |  | | |  | |  | | |  | | 0.0079 | | | 1.75 | |  | |
| NM_007904 | | | Ednrb | | | Mus musculus endothelin receptor type B (Ednrb) | | |  | | |  | |  | | |  | | 0.0337 | | | 1.74 | |  | |
| NM_009306 | | | Syt1 | | | Mus musculus synaptotagmin I (Syt1) | | |  | | |  | |  | | |  | | 0.0079 | | | 1.74 | |  | |
| NM_019811 | | | Acss2 | | | Mus musculus acyl-CoA synthetase short-chain family member 2 (Acss2) | | |  | | |  | |  | | |  | | 0.0275 | | | 1.74 | |  | |
| AK050570 | | | Irf3 | | | Mus musculus interferon regulatory factor 3 | | |  | | |  | |  | | |  | | 0.0359 | | | 1.73 | |  | |
| AK172642 | | |  | | | Mus musculus Gm4289 predicted gene 4289 | | |  | | |  | |  | | |  | | 0.0173 | | | 1.72 | |  | |
| NM_181418 | | | Ushbp1 | | | Mus musculus Usher syndrome 1C binding protein 1 (Ushbp1) | | |  | | |  | |  | | |  | | 0.0079 | | | 1.70 | |  | |
| NM_026730 | | | Gpihbp1 | | | Mus musculus GPI-anchored HDL-binding protein 1 (Gpihbp1) | | |  | | |  | |  | | |  | | 0.0000 | | | 1.69 | |  | |
| NM_007843 | | | Defb1 | | | Mus musculus defensin beta 1 (Defb1) | | |  | | |  | |  | | |  | | 0.0000 | | | 1.69 | |  | |
| ENSMUST00000102582 | | | Acacb | | | Mus musculus Acacb acetyl-Coenzyme A carboxylase beta | | |  | | |  | |  | | |  | | 0.0079 | | | 1.68 | |  | |
| NM_026531 | | | Isg20l1 | | | Mus musculus interferon stimulated exonuclease gene 20-like 1 (Isg20l1) | | |  | | |  | |  | | |  | | 0.0000 | | | 1.68 | |  | |
| NM_024198 | | | Gpx7 | | | Mus musculus glutathione peroxidase 7 (Gpx7) | | |  | | |  | |  | | |  | | 0.0000 | | | 1.67 | |  | |
| NM_007988 | | | Fasn | | | Mus musculus fatty acid synthase (Fasn) | | |  | | |  | |  | | |  | | 0.0145 | | | 1.66 | |  | |
| NM_010612 | | | Kdr | | | Mus musculus kinase insert domain protein receptor (Kdr) | | |  | | |  | |  | | |  | | 0.02 | | | 1.66 | |  | |
| NM_153288 | | | Npb | | | Mus musculus neuropeptide B (Npb) | | |  | | |  | |  | | |  | | 0.0449 | | | 1.66 | |  | |
| AK140938 | | | 1810026B05Rik | | | Mus musculus 1810026B05Rik RIKEN cDNA 1810026B05 gene | | |  | | |  | |  | | |  | | 0.0412 | | | -1.66 | |  | |
| NAP030391-1 | | |  | | |  | | |  | | |  | |  | | |  | | 0.0173 | | | -1.66 | |  | |
| AK122234 | | | Rapgef5 | | | Mus musculus Rapgef5 Rap guanine nucleotide exchange factor (GEF) 5 | | |  | | |  | |  | | |  | | 0.0449 | | | 1.65 | |  | |
| NM_010681 | | | Lama4 | | | Mus musculus laminin, alpha 4 (Lama4) | | |  | | |  | |  | | |  | | 0.0260 | | | 1.64 | |  | |
| NM_016884 | | | Hnrpc | | | Mus musculus heterogeneous nuclear ribonucleoprotein C (Hnrpc) | | |  | | |  | |  | | |  | | 0.0359 | | | -1.64 | |  | |
| NM_020574 | | | Kcne3 | | | Mus musculus potassium voltage-gated channel, Isk-related subfamily, gene 3 (Kcne3) | | |  | | |  | |  | | |  | | 0.0000 | | | 1.64 | |  | |
| NM_026278 | | | Lrp2bp | | | Mus musculus Lrp2 binding protein, mRNA (cDNA clone IMAGE:4504268), partial cds [BC030908] | | |  | | |  | |  | | |  | | 0.0000 | | | 1.63 | |  | |
| NM_172050 | | | Cd300e | | | Mus musculus CD300e antigen (Cd300e), mRNA [NM_172050] | | |  | | |  | |  | | |  | | 0.0079 | | | 1.63 | |  | |
| NM_028544 | | | Rasip1 | | | Mus musculus Ras interacting protein 1 (Rasip1), mRNA [NM_028544] | | | 0.0438 | | | 1.28 | |  | | |  | | 0.0000 | | | 1.63 | |  | |
| NM_020026 | | | B3galnt1 | | | Mus musculus UDP-GalNAc:betaGlcNAc beta 1,3-galactosaminyltransferase, polypeptide 1 (B3galnt1) | | |  | | |  | |  | | |  | | 0.0275 | | | -1.63 | |  | |
| AK002213 | | | Gstm7 | | | Mus musculus Gstm7 glutathione S-transferase, mu 7 | | |  | | |  | |  | | |  | | 0.0337 | | | 1.62 | |  | |
| NM_178061 | | | Mobkl2b | | | Mus musculus MOB1, Mps One Binder kinase activator-like 2B (yeast) (Mobkl2b) | | |  | | |  | |  | | |  | | 0.0133 | | | 1.61 | |  | |
| NM_178928 | | | AI173486 | | | Mus musculus expressed sequence AI173486 (AI173486) | | |  | | |  | |  | | |  | | 0.0211 | | | 1.61 | |  | |
| NM_007843 | | | Defb1 | | | Mus musculus defensin beta 1 (Defb1) | | |  | | |  | |  | | |  | | 0.0000 | | | 1.61 | |  | |
| NM_027209 | | | Ms4a6b | | | Mus musculus membrane-spanning 4-domains, subfamily A, member 6B (Ms4a6b) | | |  | | |  | |  | | |  | | 0.0337 | | | 1.61 | |  | |
| CJ243128 | | |  | | | unknown EST | | |  | | |  | |  | | |  | | 0.0133 | | | -1.61 | |  | |
| NM_008361 | | | Il1b | | | Mus musculus interleukin 1 beta (Il1b) | | |  | | |  | |  | | |  | | 0.0359 | | | 1.60 | |  | |
| NM_011022 | | | Ott | | | Mus musculus ovary testis transcribed (Ott) | | |  | | |  | |  | | |  | | 0.0359 | | | -1.60 | |  | |
| NM_009672 | | | Anp32a | | | Mus musculus acidic (leucine-rich) nuclear phosphoprotein 32 family, member A (Anp32a) | | |  | | |  | |  | | |  | | 0.0173 | | | -1.60 | |  | |
| NM_023438 | | | Tmem132e | | | Mus musculus transmembrane protein 132E (Tmem132e) | | |  | | |  | |  | | |  | | 0.0000 | | | 1.59 | |  | |
| AK033959 | | | Lpin2 | | | Mus musculus adult male diencephalon cDNA, RIKEN full-length enriched library,lipin 2 | | |  | | |  | |  | | |  | | 0.0449 | | | 1.58 | |  | |
| NM_177027 | | | Zcchc7 | | | Mus musculus zinc finger, CCHC domain containing 7 (Zcchc7) | | |  | | |  | |  | | |  | | 0.0211 | | | 1.58 | |  | |
| NM_027500 | | | 4933434E20Rik | | | Mus musculus RIKEN cDNA 4933434E20 gene, transcript variant 2 | | |  | | |  | |  | | |  | | 0.0433 | | | -1.57 | |  | |
| NM_009517 | | | Zmat3 | | | Mus musculus zinc finger matrin type 3 (Zmat3) | | |  | | |  | |  | | |  | | 0.0000 | | | 1.57 | |  | |
| NM_026271 | | | 1110018M03Rik | | | Mus musculus RIKEN cDNA 1110018M03 gene (1110018M03Rik) | | |  | | |  | |  | | |  | | 0.0173 | | | -1.56 | |  | |
| NM_008420 | | | Kcnb1 | | | Mus musculus potassium voltage gated channel, Shab-related subfamily, member 1 (Kcnb1) | | |  | | |  | |  | | |  | | 0.0412 | | | 1.55 | |  | |
| NM_028454 | | | Tm7sf2 | | | Mus musculus transmembrane 7 superfamily member 2 (Tm7sf2) | | |  | | |  | |  | | |  | | 0.0133 | | | 1.54 | |  | |
| NM_207685 | | | Elavl2 | | | Mus musculus ELAV (embryonic lethal, abnormal vision, Drosophila)-like 2 (Hu antigen B) (Elavl2), transcript variant 1 | | |  | | |  | |  | | |  | | 0.0275 | | | -1.54 | |  | |
| XM_194371 | | | Dchs1 | | | PREDICTED: Mus musculus dachsous 1 (Drosophila) (Dchs1), mRNA [XM_194371] | | |  | | |  | |  | | |  | | 0.0000 | | | 1.54 | |  | |
| TC1697186 | | |  | | | Q9C171 (Q9C171) Non-catalytic protein 1, partial (4%) [TC1434594] | | |  | | |  | |  | | |  | | 0.0248 | | | 1.54 | |  | |
| NM_177649 | | | BC034076 | | | Mus musculus cDNA sequence BC034076 (BC034076) | | |  | | |  | |  | | |  | | 0.0173 | | | 1.54 | |  | |
| NM_183405 | | | Cox6b2 | | | Mus musculus cytochrome c oxidase subunit VIb polypeptide 2 (Cox6b2) | | |  | | |  | |  | | |  | | 0.0000 | | | 1.54 | |  | |
| NM_021557 | | | Rdh11 | | | Mus musculus retinol dehydrogenase 11 (Rdh11) | | |  | | |  | |  | | |  | | 0.0133 | | | 1.53 | |  | |
| NM_013476 | | | Ar | | | Mus musculus androgen receptor (Ar) | | |  | | |  | |  | | |  | | 0.0359 | | | 1.53 | |  | |
| NM_011199 | | | Pthr1 | | | Mus musculus parathyroid hormone receptor 1 (Pthr1) | | |  | | |  | |  | | |  | | 0.0079 | | | 1.53 | |  | |
| AK136375 | | | 9230114K14Rik | | | Mus musculus 9230114K14Rik RIKEN cDNA 9230114K14 gene | | |  | | |  | |  | | |  | | 0.0000 | | | 1.53 | |  | |
| NM_028766 | | | Tmem43 | | | Mus musculus transmembrane protein 43 (Tmem43) | | |  | | |  | |  | | |  | | 0.0000 | | | 1.52 | |  | |
| NM_011609 | | | Tnfrsf1a | | | Mus musculus tumor necrosis factor receptor superfamily, member 1a (Tnfrsf1a) | | |  | | |  | |  | | |  | | 0.02 | | | 1.52 | |  | |
| AF217545 | | | Copg2as2 | | | Mus musculus Copg2as2 coatomer protein complex, subunit gamma 2, antisense 2 | | |  | | |  | |  | | |  | | 0.0275 | | | 1.52 | |  | |
| BB160563 | | |  | | | BB160563 RIKEN full-length enriched, 16 days neonate thymus Mus musculus | | |  | | |  | |  | | |  | | 0.0000 | | | -1.52 | |  | |
| NM_026764 | | | Gstm4 | | | Mus musculus glutathione S-transferase, mu 4 (Gstm4) | | |  | | |  | |  | | |  | | 0.0000 | | | 1.51 | |  | |
| NM_025956 | | | 1700011H14Rik | | | Mus musculus RIKEN cDNA 1700011H14 gene | | |  | | |  | |  | | |  | | 0.0079 | | | 1.51 | |  | |
| AK129325 | | | Hecw2 | | | Mus musculus Hecw2 HECT, C2 and WW domain containing E3 ubiquitin protein ligase 2 | | |  | | |  | |  | | |  | | 0.0309 | | | 1.51 | |  | |
| AK088732 | | | Kalrn | | | Mus musculus Kalrn kalirin, RhoGEF kinase | | |  | | |  | |  | | |  | | 0.0079 | | | -1.51 | |  | |
| AK084358 | | | Dleu2 | | | Mus musculus Dleu2 deleted in lymphocytic leukemia, 2 | | |  | | |  | |  | | |  | | 0.0495 | | | -1.51 | |  | |
| CB588406 | | |  | | | AGENCOURT_12600125 NIH_MGC_136 Mus musculus cDNA clone IMAGE:30290747 5' | | | 0.0000 | | | -1.53 | |  | | |  | |  | | |  | |  | |
| NM_018738 | | | Igtp | | | Mus musculus interferon gamma induced GTPase (Igtp) | | | 0.0000 | | | -1.97 | |  | | |  | |  | | |  | |  | |
| NM_015783 | | | Isg15 | | | Mus musculus ISG15 ubiquitin-like modifier (Isg15) | | | 0.0000 | | | -1.81 | |  | | |  | |  | | |  | |  | |
| AK162955 | | | Gemin5 | | | Mus musculus Gemin5 gem (nuclear organelle) associated protein 5 | | | 0.0000 | | | 1.64 | |  | | |  | |  | | |  | |  | |
| NM_146006 | | | Lss | | | Mus musculus lanosterol synthase | | |  | | |  | | 0.0361 | | | 1.57 | |  | | |  | |  | |
